# Omphaloceles in combination with a self-made hemispherical cushion: a report of 12 cases

**DOI:** 10.1590/1414-431X20187380

**Published:** 2018-07-30

**Authors:** Guoxian Huang, Lili Ma, Yonglong Wu

**Affiliations:** Pediatric Surgery of Xiamen Maternal and Child Health Hospital, Xiamen, Fujian, China

**Keywords:** Giant omphalocele, Non-surgical treatment in phase II, Treatment in stages, Staged treatment

## Abstract

The aim of this study was to discuss the curative effect of applying “capsule-reserved normal saline bag and self-made hemi-spherical cushion oppression” for treating giant omphaloceles. Twelve patients with giant omphaloceles who were admitted to our hospital between January 2008 and June 2016 were selected for treatment as follows: a capsule-reserved normal saline bag was used to promote the gradual return of the abdominal contents into the abdominal cavity in phase I, and a self-made hemi-spherical cushion was used for compression combined with a local dressing change in phase II to treat the giant omphaloceles without surgical treatment. All 12 patients in this group were cured, and after follow-up visits for >10 months, they had no abdominal infections, wound disruption, intestinal obstruction, or other complications, and their growth was normal. Two patients had abdominal hernias, and they recovered after herniorrhaphies. Giant omphaloceles in newborns were treated in stages, and in phase II, non-surgical treatment was applied, which was easily performed with a smaller wound, low cost, an obvious curative effect, and higher safety and effectiveness.

## Introduction

An omphalocele is a congenital deformity in which the abdominal organs bulge from the body caused by defects of the abdominal wall due to hypoplasia around the umbilical cord. Based on international statistics, the occurrence rate is 1–2.5 per 5,000 live births (1). Live birth means a baby who has at least one of the three life phenomena of breathing, heartbeat, and voluntary muscle twitching after exiting the mother's body. Some scholars, such as Towne, et al. (2) defined an omphalocele with liver within the bulged content as a giant omphalocele. The abdominal wall defect in a giant omphalocele is >5 cm, and is accompanied by multiple malformations. If not treated in a timely fashion, the fatality rate is very high. Currently, the main treatment methods for giant omphalocele include use of a silica gel bag, silo bag, or umbilical cord (3) for hanging, and then repair or staged surgery at a later time.

Here, we report 12 cases of patients with giant omphaloceles who were admitted to our hospital between January 2008 and June 2016. Patients were treated with a capsule-reserved normal saline bag and a non-surgical method involving a self-made hemi-spherical cushion oppression to gradually return the organs into the abdomen, obtaining a satisfactory outcome.

## Material and Methods

### General data

Twelve patients were included in the study (9 males and 3 females). At the time of admission to our hospital, the patient ages were 1–14 h, with an average age of 5 h. The birth weights ranged from 1830 to 3150 g, with an average weight of 2660 g. Four patients had atrial septal defects, 4 had cryptorchidism, 4 had indirect inguinal hernias, and 2 had hypospadias. The minimum and maximum bulges were 9 × 8 × 7 and 10 × 11 × 9 cm, respectively. The minimum and maximum basal defects were 5 × 5 and 7 × 6 cm, respectively. None of the patients had capsule ruptures or exposure of abdominal organs; the bulging content included the liver and intestinal canal, and all had meconium defecation from the anus after birth.

### Treatment

All of the patients underwent emergency surgery in phase I. The patients were administered ketamine intravenously and the capsules were gently flushed with normal saline and 0.5% iodophor, and were not damaged. After disinfection, a normal saline bag (disinfected) was used for temporary suturing of the abdominal wall around the capsule to form a capsular bag. In general, 6–8 stitches were needed, and the capsular bag was compressed to promote the return of the abdominal contents. The periphery on the bottom of the saline bag was wrapped with dry gauze. After undergoing surgery, the patients were placed in an incubator or re-warming platform and the capsular bag was hung to avoid compressing the patient's abdominal cavity and affecting cardio-pulmonary function. Post-operatively, 0.5% iodophor was injected into the capsular bag daily, and the gauze around the bag was replaced regularly. Beginning 2–3 days post-operatively, the capsular bag was compressed every day and tied with double-strand 2–0 thread to gradually enlarge the abdominal cavity and return the organs into the cavity. The compression did not affect the patient's respiration or caused edema of the lower limbs; defecation and gastrointestinal decompression status were observed. If the patient defecated without difficulty and had a small gastrointestinal decompression, he/she could resume feeding gradually.

**Figure 1.**
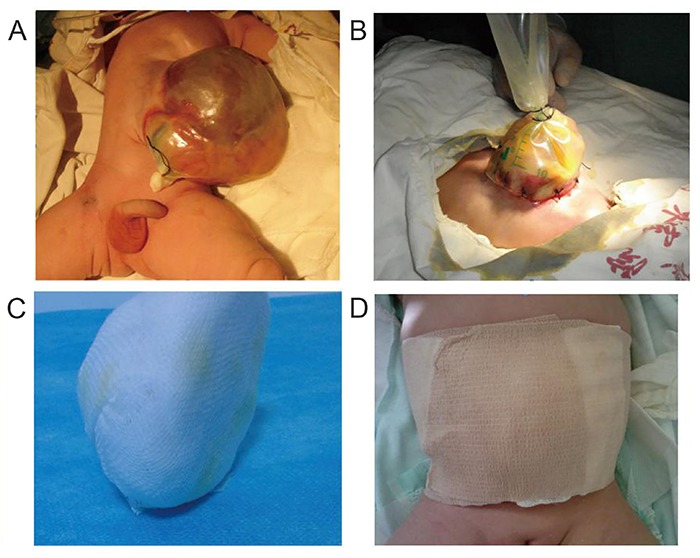
Local compression with self-made hemi-spherical gauze cushion to promote return of bulged organs into the abdominal cavity. *A*: A patient with giant omphalocele; *B*: Omphalotaxis with silica gel bag in phase I; *C*, *D*: Local compression with self-made hemi-spherical gauze cushion and elastic bellyband to promote the return of organs.

The organs in the capsules returned to the abdominal cavity 7–8 days post-operatively in most patients. The capsular bag was then removed, which revealed necrosis and drying of the surface capsule. The necrotic and dried capsule was removed daily, and after disinfection of the wound surface with 0.5% iodophor, the wound was covered with petrolatum gauze to prevent adhesions. A self-made hemi-spherical gauze cushion and elastic bellyband (Figure 1) was used for local compression to promote the return of the bulged content. Daily outpatient dressing changes and disinfection were performed, and the necrotic capsule was gradually removed. The defects grew naturally; thus, there was little influence on respiration and circulation, and the feeding was not affected. Post-operatively, the patients remained in the hospital for an average of 18 days; the patients were discharged early to recover more quickly.

### Post-discharge treatment and follow-up

After discharge, the patients were continuously disinfected and bound daily. The patients were transported to our hospital for follow-up visits until the umbilical defects were completely healed. The post-operative skin healing time was 1–2 months, with an average length of 1 month and 11 days. The defects completely healed spontaneously, and the umbilicus formed naturally. We telephoned each patient on a regular basis to determine the feeding and defecation status and the presence of complications, such as intestinal obstruction and abdominal hernia.

## Results

All 12 patients were visited for 10 months to 6 years and 5 months during follow-up, with an average duration of follow-up of 3 years and 8 months (Table 1). All patients recovered without complications, were following normal diets, and defecating regularly. There was no report of abdominal infections, wound disruption, intestinal obstruction, or other complications, and the growth trajectories were normal with normally formed umbilici. Two patients had abdominal hernias, and underwent herniorrhaphies at 1 year of age; they recovered post-operatively without recurrences.

**Table 1.** Characteristics of 12 patients with giant umbilical bulging.

No.	Gender	Age (h)	Weight (g)	Apgar (points)	Abdominal wall defect size (cm × cm)	Associated malformation	Surgery complications	Follow-up time (months)
1	Male	4.5	2560	8–10–10	6 × 5	Atrial septal defect / Inguinal hernia	Abdominal hernia	77
2	Male	10	2850	10–10–10	6.5 × 5	Inguinal hernia		72
3	Male	8	2960	10–10–10	6 × 5	Cryptorchidism		70
4	Male	1	2150	7–9–10	5 × 5	Atrial septal defect / Cryptorchidism		66
5	Male	3	2380	10–10–10	5.5 × 5	Hypospadias / cryptorchidism		57
6	Male	2	3050	10–10–10	7 × 6		Abdominal hernia	46
7	Female	14	2230	9–10–10	6 × 5	Atrial septal defect		37
8	Male	2	3150	10–10–10	6 × 6	Inguinal hernia		32
9	Male	6	2710	10–10–10	6 × 5			26
10	Female	3.5	2460	8–9–10	5 × 5	Atrial septal defect		18
11	Female	4	2610	10–10–10	5 × 5	Cryptorchidism		16
12	Male	2	2980	10–10–10	6 × 5	Hypospadias / Inguinal hernia		10

## Discussion

### Current trends in the domestic and international treatment of giant omphaloceles

Currently, repair in phase I and staged repairs are the main methods of giant omphalocele treatment. Silica gel, silo, or blood bags (4) or umbilical cords (5) are used first for hanging in phase I to promote the gradual return of the bulged content. After approximately 1 week, a delayed repair or staged surgery of the abdominal wall defect repair is performed.

**Figure 2.**
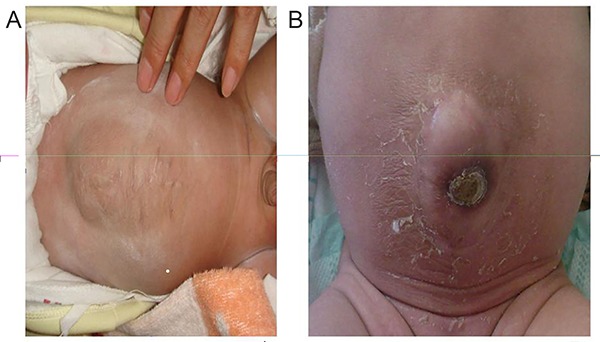
Comparison of effect on healing via operative treatment (*A*), and local compression (*B*) in phase II.

Since Schuster first treated the gastroschisis successfully with staged repair surgery in 1967, staged reduction as early as possible has been recommended in the treatment of children with giant omphaloceles and no capsule rupture (6–9). In phase I, a silica gel bag or a capsular bag made of polyester fabric is used to wrap and hang the bulged contents. The capsular bag is tightened in volume daily to promote the return of the organs. Without compression of the capsular bag, the return of bulged contents is gradually achieved by hanging and organ gravity alone (10). Abdominal wall repair surgery is then conducted in phase II. During surgery, the bulged contents are returned completely; however, due to the large abdominal defect, the abdominal tension is high after suturing the abdominal wall, greatly affecting respiration and circulation. The patient often needs assisted ventilation with a respirator after surgery, and feeding is delayed, requiring total intravenous nutrition for a long time. In addition, due to dysplasia of the abdominal wall layers, an imbalance in tensions occurs. Moreover, the incision is irregular in shape, which will not affect the physiologic function, but can affect the appearance of the umbilicus post-operatively (Figure 2).

The advantage of this method is that through surgery in phase II, intraperitoneal malformations, such as diaphragmatic hernias and intestinal malformation, can be detected and treated at the same time.

Currently, local disinfection and capsule incrustation agents, such as 2% mercurochrome and 0.5% silver nitrate, are used for conservative treatment of those patients who come late to physician care, have poor health status and apparent complications, have a large defect (>10 cm) occupying most of the anterior abdominal wall, and an apparent local infection of bulged contents. Generally, in 2–3 months the entire capsule can be covered, after which the abdomen is bound with a multi-thread bellyband to facilitate complete return of the bulged organs to the abdominal cavity. When the budged organs are fully returned to the abdominal cavity without tension in 6–12 months, the abdominal wall defect is repaired surgically (11). The disadvantage of local conservative treatment is that due to the time elapsed, the possibility of a secondary capsule wall rupture, local infection, extended length of hospital stay, and residual abdominal hernia may occur.

### Capsule-reserved normal saline bag and self-made hemi-spherical cushion oppression combination treatment

If there are no complications, such as diaphragmatic hernias, noted during pre-operative examination in phase I, traditional-staged surgery and local conservative treatment may be combined for treatment of giant omphaloceles. In phase I, a silica gel or capsular bag made of polyester fabric is used to wrap and hang the bulged contents, to promote the return of the bulged contents, and during this period, the close monitoring of defecation and gastrointestinal decompression status is required. If the patient has adequate defecation and minimal gastrointestinal decompression, the intestinal tract is not affected by intestinal atresia, intestinal malrotation, or other complications. At this time, the patient can be fed slowly, and a hemi-spherical gauze roll and an elastic bellyband are used for local compression to promote the further return of bulged contents. The patient is disinfected in the outpatient service daily to promote the natural growth of the local defect after removal of the capsule.

After rapid return of the bulged contents (7–8 days) in phase I, local compression and dressing changes are continued to promote the autogenous healing and growth of the defect. The duration of local conservative treatment are shortened (the defects in the 12 patients healed in approximately 40 days after birth). The bulged contents are returned quickly with local compression, and the abdominal muscles healed quickly, which avoided the formation of abdominal hernias caused by skin coverage (only 2 patients had abdominal hernias after surgery).

This method has nearly no influence on respirations and circulation, and does not require mechanical ventilation. Moreover, the patient can be fed early, the parenteral nutrition and antibiotic use can be reduced, and hospitalization expenses are less. In addition, the abdominal wall will have no incision after autogenous healing of the defect, and the umbilical region appears natural (Figure 2B).

Based on the size of the umbilical defect, medical gauze is wrapped to form a hemi-spherical solid gauze roll cushion slightly larger than the umbilicus in diameter (the cushion diameter is consistent with the umbilical defect; Figure 1C). Before use of this cushion, the wound surface needs a thorough debridement. Then, the wound is covered with petrolatum gauze to prevent adhesions. The center of the cushion compress the umbilical defect by an elastic bellyband (Figure 1D). The principle of such treatment is still unknown, and it may be similar to that of autogenous healing of the umbilical hernia and umbilical ring defect. With pressurization of the gauze roll cushion and elastic bellyband, maximum healing of the muscle around the umbilical defect occurred, thus avoiding the formation of an abdominal hernia caused only by skin covering.

### Cautions of combined treatment

Acromphalus is often combined with other developmental malformations; the reported occurrence rate is >50% (12). The abdominal cavity cannot be assessed with this method, so this research only applies to the patients with giant acromphalus that is not accompanied by intestinal atresia, intestinal malrotation, and other complications. Therefore, in the hanging process of phase I, it is necessary to observe the gastrointestinal decompression and defecation status. Four of our patients with giant acromphaluses had gastrointestinal decompression in the hanging process of phase I. The color of contents from the gastrointestinal decompression was yellowish green and the amount was relative large. We considered that the giant acromphaluses were accompanied by digestive tract developmental malformations, so we obtained digestive tract studies, which showed intestinal malrotation. Thus, we performed surgery for return of organs in phase II, during which intestinal malrotation was confirmed.

During early dressing changes, 10% chloral hydrate (0.5 mL/kg) is administered to help calm the patient and avoid further injury to the bulge of abdominal organs due to agitation and sobbing. It is also necessary to remove the dried and necrotic outer capsule from the periphery to keep the wound surface in a fresh granulation state, so that the surrounding skin cells can cover the wound.

During the suturing and hanging in phase I, infection may occur after 7 days. With infection, the normal saline bag is torn from the surrounding tissues under the action of tension, resulting in surgical failure (13). In this group of patients, the following treatment was administered to prevent infections: i) broad-spectrum antibiotics intravenously, and 0.5% iodophor injected to the capsular bag every day; ii) after strict disinfection during surgery in phase I, the capsule should be retained and even the broken capsule should be repaired, if possible, and reserved; iii) the artificial capsular bag was wrapped with several layers of wet gauze and normal saline to keep the water and drain the abdominal effusion, then it was wrapped with an aseptic dressing to reduce the contact of effusion and the outside; iv) the dressing was changed when the capsular bag was shrunk every day, and the effusion property change was observed; and v) bacterial culture was done when necessary. When the capsule was dried and pressure dressing was applied in phase II, the wound surface was disinfected during each dressing change; dried and the necrotic tissue was removed promptly. In this group of patients, no infections were recorded.

During treatment, it is necessary to apply the pressure dressing under proper intra-abdominal pressure and follow the principle of “better loose than tight,” to avoid influence on respiration. Intravesical pressure measurement is the best method for determining intra-abdominal pressure. According to the World Society of the Abdominal Compartment Syndrome (WSACS), adult intra-abdominal hypertension is divided into 4 grades based on the method of intravesical pressure measurement, while there in no indicator for newborns. It has been reported (14) that before and after the return of organs and abdominal defect tensioning, the intravesical pressure increase should not exceed 10 cm H_2_O, the increase in respiratory rate should not exceed 4 breath/min, and the increase in heart rate should not exceed 15 beats/min, to avoid causing abdominal compartment syndrome due to a rapid return of organs.

In conclusion, the data showed that after combined treatment with a “capsule-reserved normal saline bag for repair in phase I and self-made hemi-spherical cushion compression,” the 12 patients achieved a good outcome, with minimal trauma and low treatment expenses. We have described a new treatment method for giant omphaloceles and believe that further research is warranted.
